# Effects of acupuncture on serum metabolic parameters in premenopausal obese women: study protocol for a randomized controlled trial

**DOI:** 10.1186/s13063-015-0867-y

**Published:** 2015-08-04

**Authors:** Koh-Woon Kim, Hye Hyun Yoo, Jae-Heung Cho, Yo-Chan Yang, Je-In Kim, Song-Yi Kim, Ji-Yeun Park, Hi-Joon Park, Mi-Yeon Song

**Affiliations:** Department of Korean Rehabilitation Medicine, College of Korean Medicine, Kyung Hee University, 26 Kyungheedae-ro, Dongdaemun-gu, Seoul, 130-701 Republic of Korea; Institute of Pharmaceutical Science and Technology and College of Pharmacy, Hanyang University, Ansan-si, Gyeonggi-do 426-791 Republic of Korea; Studies of Translational Acupuncture Research, Acupuncture and Meridian Science Research Center, Kyung Hee University, 26 Kyungheedae-ro, Dongdaemun-gu, Seoul, 130-701 Republic of Korea; Department of Acupoint and Meridian, College of Korean Medicine, Kyung Hee University, 26 Kyungheedae-ro, Dongdaemun-gu, Seoul, 130-701 Republic of Korea

**Keywords:** Obesity, Acupuncture, Metabolomics, Effect, Safety, Clinical research protocol

## Abstract

**Background:**

Complex metabolic changes cause obesity, making weight loss difficult. For this reason, understanding metabolism is important, and considering the shortcomings of conventional treatment options for obesity, acupuncture is a possible option. However, evidence supporting its efficacy on metabolic parameters in obese patients is lacking. The aim of this study is to investigate the effects of acupuncture on serum metabolic parameters in premenopausal obese women.

**Methods/design:**

This ongoing study is a randomized, patient-assessor blind, two-arm parallel non-penetrating sham-controlled clinical trial. Eligible participants, premenopausal adult women (19 years of age or older) with a clinical diagnosis of obesity (body mass index of 25 kg/m^2^ or more) blinded to the treatment received, will be randomly allocated blindly into the real acupuncture treatment group (manual acupuncture plus electroacupuncture, n = 60) or the sham acupuncture control group (sham acupuncture plus placebo acupuncture without electrical stimulation, n = 60) and receive treatment two times a week for a total of 12 sessions over 6 weeks. The primary outcome measure is the serum cholesterol and triglyceride (TG) levels at baseline and endpoint. The secondary outcomes are body weight, body fat mass, muscle mass, waist and hip circumference, other serum metabolic profiles, International Physical Activity Questionnaire (IPAQ), Social Readjustment Rating Scale (SRRS), Stress Response Inventory (SRI), Fatigue Severity Scale (FSS), the Korean version of the Beck Depression Inventory (BDI), and urine metabolites. Adverse events will be assessed at every visit.

**Discussion:**

The results of this trial (which will be available in 2015) will provide important clinical evidence for the effect of acupuncture on serum metabolites and demonstrate how acupuncture can be helpful for the treatment of obesity.

**Trial registration:**

Trial registration registered via US National Institutes of Health Clinical Trials registry (ClinicalTrials.gov) on 11 November 2014, identifier: NCT02066090.

## Background

Obesity is a major health problem associated with morbidity (for example, type 2 diabetes mellitus, certain cancers, and cardiovascular and musculoskeletal diseases) and premature death [1–4]. Treatment options include dietary and lifestyle changes, drug interventions, and bariatric surgery [5]. The UK National Institute for Health and Clinical Excellence (NICE) advises that lifestyle changes, including behavioral approaches, should form the mainstay of obesity management [6]. However, many studies of dietary and behavioral treatments showed that maintaining weight loss is difficult when obesity is associated with various metabolic changes [7] and caused by complex interactions between environment, genetic predisposition, and personal behavior [8]. Recently, investigators have applied comprehensive metabolomic profiling to gain a better understanding of biochemical, endocrine, inflammatory marker, and physiological differences between obese and lean human subjects [9, 10].

Given the shortcomings of conventional treatment options and the importance of understanding metabolism in obesity, acupuncture is a possible treatment option for obese patients. Three systematic reviews of randomized controlled trials have shown the benefits of acupuncture in patients with obesity [11–13]. The results were promising, but many of the trial components were of poor quality. Several studies showed that acupuncture treatment may specifically aid in weight loss and reduce obesity by stimulating alpha-melanocyte-stimulating hormone expression and release [14], enhancing the expression of peroxisome proliferator-activated receptor gamma mRNA in adipose tissues [15], reducing blood lipids and fasting blood glucose levels [16], and upregulating obestatin expression in the hypothalamus [17]. These results suggested possible mechanisms of acupuncture for obesity but were inconclusive because they involved experiments using animal models. A few clinical trials have examined the effect of acupuncture on metabolic parameters in obese patients [18].

In this paper, we propose a randomized, patient-assessor blind, sham-controlled study of patients with obesity, with a predetermined sample size and appropriate follow-up, which will help to investigate the efficacy of acupuncture on obesity from the perspective of metabolomics and therefore provide a better understanding of the physiological therapeutic mechanism of acupuncture on obesity.

## Methods/design

### Study design

**Fig. 1:**
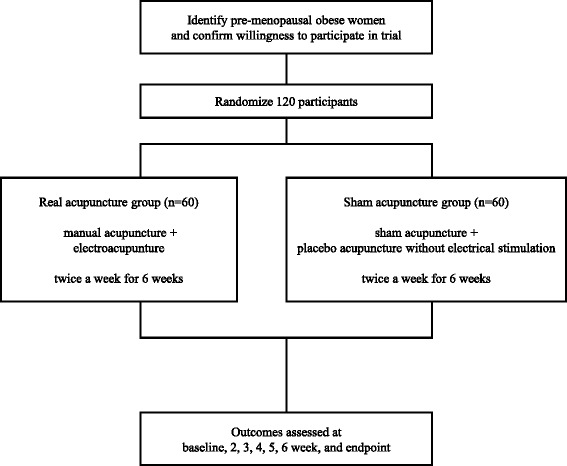
The study flowchart

This study is a two-arm parallel, patient-assessor blinded, non-penetrating sham-controlled randomized clinical trial. The trial will be performed in the Korean Medicine Hospital of Kyung Hee University at Gangdong in Korea in accordance with the Declaration of Helsinki and the Guidelines for Good Clinical Practice. This protocol was registered with the US National Institutes of Health Clinical Trials registry. Eligible participants will be randomly allocated into one of two groups (the real acupuncture treatment group or the sham acupuncture control group) in a 1:1 allocation ratio, blinded to the group allocation, and will receive treatment for 6 weeks (Fig. 1). The outcome assessment and the statistical analysis will be performed by professionals blinded to the assignment of participants to either the real or sham acupuncture.

### Participants

#### Inclusion criteria

A total of 120 patients will be recruited through advertisements posted in local newspapers, in the hospital’s monthly magazine, on a website, and on bulletin boards. If patients are interested in participating, they will be asked to answer questions to determine eligibility. The eligibility criteria for the study include adult women (19 years of age or older) in premenopausal state with regular menstruation, clinical diagnosis of obesity (body mass index of 25 kg/m^2^ or more), and signed written informed consent. To avoid the effect of menopause on obesity and serum metabolic parameters [19], we decided to include only premenopausal women.

#### Exclusion criteria

Participants will be excluded if they are experiencing or have a history of the following: previous or current endocrine disease such as hypothyroidism or Cushing’s syndrome, heart disease, lung disease, diabetes, malignant tumor, cholelithiasis, severe kidney impairment, severe liver impairment, anorexia nervosa or hyperorexia; history of taking medicines which might affect body weight and the concentration of serum metabolic parameters such as anorectic, laxative, oral steroid, thyroid hormone, amphetamine, cyproheptadine, and phenothiazine medicines, or medicines affecting absorption, metabolism, and excretion during the last 3 months; history of taking beta blockers or diuretics for the treatment of hypertension during the last 3 months; surgical treatment for obesity; currently pregnant or breast-feeding or planning to become pregnant; history of taking other experimental medicines during the last 1 month; weight loss of 10 % or more of previous body weight during the last 6 months; stopped smoking during the last 3 months or have irregular smoking habits; pacemaker; currently receiving any treatment for obesity or other inappropriate reasons for inclusion in this trial, including unwillingness to comply with this study protocol or inability to complete the study-related questionnaires by themselves or with assistance, as assessed by researchers.

### Randomization

Study participants who meet the eligibility criteria will be randomly assigned to two groups (the real acupuncture treatment group or the sham acupuncture control group) in a 1:1 ratio at the second visit after signing written informed consent. Randomization will be conducted using a computer-generated random allocation sequence by a statistician with no clinical involvement in this trial, and the random code will be kept by a clinician who will not contact patients. Blocked randomization will be employed to ensure balance within the two groups.

Subject details will be recorded, and a treatment arm and randomization number will be allocated to the subject. Randomization code will be released after the participants are recruited for the trial, and all baseline measures are taken to ensure allocation concealment. The practitioners will be aware of the allocation arm, while the subjects, outcome assessors, and statistician performing the data analysis will be blinded to the treatment allocation [20]. The blinding credibility of the real and sham acupuncture treatments will be evaluated at baseline and at the end of the 6-week treatment [21].

### Intervention

Both real and sham acupuncture groups will receive a total of 10–12 acupuncture sessions. At the time of the first acupuncture session, all participants will be given a “dietary and exercise manual for obese patients” from the obesity clinic of the Korean Medicine Hospital of Kyung Hee University at Gangdong.

#### Real acupuncture treatment group

The real acupuncture group will receive manual acupuncture plus electroacupuncture treatment at the same time twice a week for 6 weeks; these are common approaches used by Korean medicine doctors (KMDs) in South Korea today. The predefined acupuncture points were carefully selected by a process of consensus with participating KMDs who all specialize in obesity treatment on the basis of a literature review regarding acupuncture for obesity. The 14 acupuncture points used were the following: bilateral Hegu (LI4), Quchi (LI11), Sanyinjiao (SP6), Zusanli (ST36), middle Qihai (CV6), and Zhongwan (CV12) for manual acupuncture and bilateral Tianshu (ST25) and Shuidao (ST28) for electroacupuncture.

Manual acupuncture treatment will be administered using disposable sterile stainless steel needles (40 mm × 0.25 mm; Dong-bang Acupuncture Inc., Seoul, Korea) to the 10 acupuncture points mentioned above with the aid of the tube of a sham acupuncture needle device. The needles will be inserted perpendicularly to a depth of 5–20 mm, with the patient lying down and blindfolded after skin sterilization, followed by bidirectional rotation to induce *deqi* sensation. The needles will then be left in place for 30 minutes.

At the same time, electroacupuncture treatment will be administered using disposable sterile stainless steel needles (90 mm × 0.25 mm; Dong-bang Acupuncture Inc.) and a LipoDR low-frequency electroacupuncture device (Dow Meditec Corp., Seoul, Korea) to the above-mentioned 4 abdominal acupuncture points. The needles will be inserted horizontally to a depth of 50–80 mm and will be connected to the LipoDR electrical stimulator. Electrical stimulation will consist of biphasic constant current (2 mA) pulses of 25 Hz for 25 minutes followed by 60 Hz for 5 minutes.

#### Sham acupuncture control group

The sham acupuncture group will receive non-penetrating sham acupuncture as a control for manual acupuncture plus placebo acupuncture without electrical stimulation as a control for electroacupuncture at the same time twice a week for 6 weeks.

Sham acupuncture treatment will be administered using non-penetrating disposable sterile stainless steel sham needles (40 mm × 0.25 mm) as described by Park et al*.* (AcuPrime Co., Ltd., Exeter, UK) [22] to the same 10 acupuncture points as used for the real acupuncture group. Except for the usage of a semi-blunted needle, the technique will be the same as that performed in the real acupuncture group.

At the same time, placebo acupuncture treatment without electrical stimulation will be administered using disposable sterile stainless steel needles (90 mm × 0.25 mm; Dong-bang Acupuncture Inc.) and a LipoDR dummy device to the 4 predefined non-traditional abdominal acupuncture points: 2 cm lateral to Tianshu (ST25) and 2 cm lateral to Shuidao (ST28), bilaterally. The needles will be inserted horizontally to a depth of 50–80 mm and connected to the LipoDR dummy device, which is designed to have no electrical stimulation through insulation of electrodes and is left in place for 30 minutes.

#### Practitioner background

The real and sham acupuncture treatments will be conducted by KMDs licensed by the Korean Ministry of Health and Welfare who specialize in Korean rehabilitation medicine with at least 4 years of clinical experience. They will be required to take an educational course to strictly adhere to the study protocol and be familiar with administering the study treatments. All practitioners will undergo intensive and customized training for a full understanding of the “sham acupuncture” procedure and will be trained to administer real acupuncture using a sham needle device. The techniques for all the treatment procedures will be standardized among practitioners.

### Outcome

#### Primary outcome measurement

The primary outcome measure is the serum cholesterol levels including HDL cholesterol, LDL cholesterol, and triglyceride (TG) levels. Peripheral blood will be drawn from the antecubital veins of the subjects in the real and sham acupuncture groups at baseline (visit 2) and endpoint (visit 14: within 3 days after completion of the 6-week treatment) after a 10–12 hour overnight fast. Blood samples will be collected in serum-separating tubes (5 mL), sodium fluoride tubes (3 mL), and EDTA-coated tubes (2 mL) and will be centrifuged at 3,000 rpm for 10 minutes to separate either serum or plasma depending on the requirement of the analysis. Aliquots of plasma and sera will be obtained, and serum-separating tubes and sodium fluoride tubes will be stored at 2 to 10 °C while EDTA-coated tubes will be stored at −80 °C for further analysis. Analysis of TG, HDL cholesterol, LDL cholesterol, free fatty acids, glucose, hs-CRP, and insulin will be performed by an outsourcing laboratory (EOne Laboratories, Incheon, South Korea), and analysis of other lipids, amino acids, and carnitines will be commissioned to the research laboratory of the College of Pharmacy, Hanyang University in Korea.

#### Secondary outcome measurements

Body weight, body fat mass, and muscle mass will be measured by bioelectrical impedance analysis using a body composition analyzer (InBody 720, InBody Co., Ltd., Seoul, Korea) and waist and hip circumference will be measured three times by the same observer according to the World Health Organization (WHO) method [23], midpoint between the lower end of the rib cage and top of the iliac crest in a standing position, which is usually 3 cm above the anterior superior iliac spine and the widest portion of the buttocks at baseline, at 2, 3, 4, 5, and 6 weeks and endpoint (visits 2, 4, 6, 8, 10, 12, 14).

Serum metabolic profile including the following will be determined: phospholipids, fatty acids, valine, tyrosine, leucine, glycine, alanine, glutamic acid, histidine, pipecolic acid, L-carnitine, acetylcarnitine, propionylcarnitine, butyrylcarnitine, isovalerylcarnitine, hexanoylcarnitine, ocatanoylcarnitine, decanoylcarnitine, dodecanoylcarnitine, tetradeconoylcarnitine, hexadecanoylcarnitine, and octadecanoylcarnitine. Glucose, hs-CRP, and insulin will also be measured (visits 2, 14).

The International Physical Activity Questionnaire (IPAQ) [24] will be used to estimate physical activities at baseline and endpoint (visits 2, 14). IPAQ is a seven-item instrument consisting of six questions that participants answer to record the number of days (frequency) and the number of minutes per day (duration) of their participation in all types of vigorous or moderate physical activities including walking during the last 7 days. In addition, the seventh question relates to the time that participants spend sitting during an average weekday. IPAQ is considered a reasonably valid measurement tool for measuring habitual physical activities based on a recent meta-analysis [25].

The Social Readjustment Rating Scale (SRRS) [26] and the Stress Response Inventory (SRI) [27] will be used to assess stress response at baseline and endpoint (visits 2, 14). SRRS is a very well-known 43 life events-list scale measuring stress that affects health based on a relative score. Subsequent validation has supported the links between stress and illness [28]; however, the scale has been criticized for its limited clinical utility. SRI includes emotional, somatic, cognitive, and behavioral stress responses and has a significantly high reliability and validity in clinical practice [27].

The Fatigue Severity Scale (FSS) [29] will be used to assess fatigability at baseline, 2, 3, 4, 5, and 6 weeks, and endpoint (visits 2, 4, 6, 8, 10, 12, 14). FSS is the most commonly used 9-item self-report questionnaire to measure fatigue [30] and was validated as a simple and reliable instrument to assess and quantify fatigue for clinical and research purposes [31].

The Korean version of Beck’s Depression Inventory (BDI) [32] will be used to measure depressive symptoms at baseline and endpoint (visits 2, 14). BDI is a 21-item self-administered questionnaire with each item having a 0–3 response format and a maximum theoretical score of 63. The psychometric quality of this test is very good [33].

Urine samples will be measured at baseline and endpoint (visits 2, 14). Liquid chromatography quadrupole time-of-flight mass spectrometry-based nontargeted metabolomic analysis will be conducted with urine and serum samples.

#### Other assessment measures

The validated Korean version of the credibility test, which was first proposed by Vincent [21], will be used to assess the credibility of real and sham acupuncture treatments at endpoint (visit 14).

An overview of the outcome measurement time points is presented in Table 1.

**Table 1:** Schedule for data collection, treatments, and outcome measurements

Period	S	T												F
Visit	1	2	3	4	5	6	7	8	9	10	11	12	13	14
Week	0	1^a^	1	2	2	3	3	4	4	5	5	6	6^b^	7^c^
Eligibility test	○													
Informed consent	○													
Demographic characteristics	○													
Random allocation		○												
Treatment		○	○	○	○	○	○	○	○	○	○	○	○	
Serum metabolites		○												○
BIA		○		○		○		○		○		○		○
WC/HC		○		○		○		○		○		○		○
IPAQ		○												○
SRRS		○												○
SRI		○												○
FSS		○		○		○		○		○		○		○
BDI		○												○
Urine metabolites		○												○
Adverse events		○	○	○	○	○	○	○	○	○	○	○	○	○
Credibility test														○

*S* screening period, *T* treatment period, *F* follow-up period, *BIA* bioelectrical impedance analysis, *WC* waist circumference, *HC* hip circumference, *IPAQ* International Physical Activity Questionnaire, *SRRS* Social Readjustment Rating Scale, *SRI* Stress Response Inventory, *FSS* Fatigue Severity Scale, *BDI* Beck’s Depression Inventory

^a^ Baseline (immediately before the first treatment); ^b^ end of 6-week treatment; ^c^ endpoint (within 3 days after completion of the 6-week treatment)

### Sample size

The following hypotheses are related to the differences between the treatment and control groups:

$$ {H}_0:{\upmu}_{\mathrm{c}}={\upmu}_{\mathrm{t}} $$

$$ {H}_1:{\upmu}_{\mathrm{c}}\ne {\upmu}_{\mathrm{t}} $$

where μ_t_ is the mean TG level in the real acupuncture treatment group and μ_c_ is the mean TG level in the sham acupuncture control group at endpoint.

Referring to a previous study [18], the researchers expect the mean difference will be 32.5 with a standard deviation of 56.907 between the two groups.

For the mean comparison method of the two-sample *t*-test model, this study will use a level of significance of α = 0.05 and a power of 1-β = 0.80 and the following two-sided test:

$$ N=\frac{2\times {\left({\mathrm{Z}}_{\upalpha}+{\mathrm{Z}}_{\upbeta}\right)}^2\times {\upsigma}^2}{\updelta^2}=\frac{2\times {\left(1.96+0.84\right)}^2\times {56.907}^2}{32.5^2}=48.07\approx 48 $$

For equal allocation of the two groups, the total sample size required when considering a dropout rate of 20 % is 120 subjects with 60 subjects in each group.

### Statistical analysis

All analyses will be performed using SPSS for Windows (version 18.0; IBM Corp., Armonk, NY, USA) and a significance level set at 0.05 by a statistician blinded to the allocation of groups. Statistical analysis will be conducted using the principles of the intention-to-treat (ITT) [34] and the per-protocol (PP) [35] analyses. We will apply the last observation carried forward (LOCF) analysis for ITT analysis.

The two-sample *t*-test for quantitative data and the chi-square test for qualitative data will be performed to test homogeneity of the baseline characteristics between the two groups. Analysis of covariance (ANCOVA) will be used for adjustment of baseline characteristics if there is a possibility of covariance. The two-sample *t*-test will be used to compare the primary and secondary variables of the two groups between baseline and endpoint. Repeated measure analysis of variance (ANOVA) will be performed for the different time point assessments between groups and interaction between groups and observed time. A mixed model approach will also be used if necessary.

### Adverse events

Any expected or unexpected adverse events will be recorded and described as frequency and percentage by the participants and practitioners at every visit to completion. The adverse events known related to acupuncture treatment include local bleeding or pain at the acupuncture points, local redness or bruising, itching, and dizziness during treatment [36]. If any serious adverse event occurs, detailed events including the date of occurrence, measures taken related to the treatment, causal relationship with the treatment, and treatment of the adverse event will be announced to the principal investigator and the institutional review board (IRB) immediately, and direct actions will be supplied to those involved.

### Ethics

The research protocol was reviewed and approved by the IRB of the Korean Medicine Hospital of Kyung Hee University at Gangdong [KHNMCOH 2013-01-020]. Written informed consent will be obtained from all participants prior to enrollment in the study.

## Discussion

So far, one clinical trial has examined the effect of acupuncture on metabolic parameters in obese patients [18], which used serum cholesterol and TG levels. Referring to the previous study, we will use serum cholesterol levels including LDL, HDL, and TG levels among serum metabolic parameters as the primary outcome measurement. We will also determine other serum metabolic profiles as secondary outcome measurements, because the main purpose of this study is to assess the efficacy of acupuncture on obesity from the perspective of metabolomics and therefore better understand the pathophysiological mechanism of obesity and acupuncture. Metabolomics is defined as a technology that aims to profile metabolite changes in response to a genetic alteration or to pathophysiological stimuli [37, 38], and it provides invaluable tools for molecular biology approaches [39]. When applied to obesity, a number of metabolic perturbations, including imbalance of glucose metabolism and dyslipidemia in key organs and blood plasma, result when one consumes a high-fat diet [7]. Thus, metabolic changes are central to obesity, and we expect that any process aimed at measuring global metabolism changes would provide good evidence for the effect of acupuncture on obesity.

We adopted a complex intervention of manual acupuncture plus electroacupuncture treatment for the real acupuncture treatment group because it is the most common approach used by KMDs in South Korea today. A review of studies on acupuncture for obesity reported that manual acupuncture and electroacupuncture are more often applied in clinical trials than auricular acupuncture and moxibustion therapy [40]. In particular, electroacupuncture was reported to decrease the serum TG, total cholesterol, and LDL cholesterol [18] and was effective on abdominal obesity [41]; thus, we decided to apply electroacupuncture on abdominal acupuncture points.

We chose non-penetrating sham acupuncture plus placebo acupuncture (non-acupuncture points) without electrical stimulation for control. In a previous study, applying sham acupuncture as a control allowed complete subject blinding [22]. Thus, sham acupuncture is considered to be a proper control for manual acupuncture. Additionally, instead of electroacupuncture on abdominal acupuncture points, we decided to use placebo acupuncture on abdominal non-acupuncture points without electrical stimulation. For valid blinding, the LipoDR dummy device was designed to have no electrical stimulation by insulation of the electrodes. The intensity of electrical stimulation from both real and dummy devices will be maintained under a certain level so that subjects in either group cannot perceive the electric current. Patients will be informed about the two types of acupuncture in the study as follows: “In this study, different types of acupuncture will be compared. The electric current will flow but with intensity under a fixed level which you cannot detect.”

In conclusion, this randomized, patient-assessor blind, sham-controlled clinical trial will provide important clinical evidence for the efficacy of acupuncture for obesity by using metabolomics methods, and will contribute to our understanding of the pathophysiological mechanisms of obesity as they relate to acupuncture.

## Trial status

The participants are currently being recruited for the present study.

## Abbreviations

ANOVA: analysis of variance
ANCOVA: analysis of covariance
BDI: Beck’s Depression Inventory
CRP: C-reactive protein
EDTA: ethylenediaminetetraacetic acid
FSS: Fatigue Severity Scale
HDL: high density lipoprotein
IPAQ: International Physical Activity Questionnaire
IRB: institutional review board
ITT: intention-to-treat
KMD: Korean medicine doctors
LDL: low density lipoprotein
LOCF: last observation carried forward
NICE: National Institute for Health and Clinical Excellence
PP: per-protocol
SPSS: Statistical Package for Social Science
SRI: Stress Response Inventory
SRRS: Social Readjustment Rating Scale
TG: triglyceride
WHO: World Health Organization
